# Preventing a Thought from Coming to Mind Elicits Increased Right Frontal Beta Just as Stopping Action Does

**DOI:** 10.1093/cercor/bhz017

**Published:** 2019-02-26

**Authors:** Anna Castiglione, Johanna Wagner, Michael Anderson, Adam R Aron

**Affiliations:** 1 Department of Psychology, University of California, San Diego, CA, USA; 2 Swartz Center for Computational Neuroscience, Institute for Neural Computation, University of California, San Diego, CA, USA; 3 MRC Cognition and Brain Sciences Unit, University of Cambridge, UK

**Keywords:** beta oscillations, inhibitory control, stop signal, think/no-think

## Abstract

In the stop-signal task, an electrophysiological signature of action-stopping is increased early right frontal beta band power for successful vs. failed stop trials. Here we tested whether the requirement to stop an unwanted thought from coming to mind also elicits this signature. We recorded scalp EEG during a Think/No-Think task and a subsequent stop signal task in 42 participants. In the Think/No-Think task, participants first learned word pairs. In a second phase, they received the left-hand word as a reminder and were cued either to retrieve the associated right-hand word (“Think”) or to stop retrieval (“No-Think”). At the end of each trial, participants reported whether they had experienced an intrusion of the associated memory. Finally, they received the left-hand reminder word and were asked to recall its associated target. Behaviorally, there was worse final recall for items in the No-Think condition, and decreased intrusions with practice for No-Think trials. For EEG, we reproduced increased early right frontal beta power for successful vs. failed action stopping. Critically, No-Think trials also elicited increased early right frontal beta power and this was stronger for trials without intrusion. These results suggest that preventing a thought from coming to mind also recruits fast prefrontal stopping.

## Introduction

The stop signal paradigm has been used to isolate the cognitive and neural mechanisms that enable people to cancel action (Logan and Cowan 1984; Verbruggen and Logan 2009). For example, using this paradigm, lesion and other disruption studies have established a critical role of the right inferior frontal cortex in action stopping (reviewed in Aron et al. 2014). Intracranial electroencephalography (EEG) recorded from this same brain region has revealed an electrophysiological signature of action stopping: increased power of beta band oscillations (~16 Hz) for successful vs. failed stopped trials within the temporal window of the inferred behavioral stopping process (Swann et al. 2009; Wessel et al. 2013). A similar right frontal signature (increased power of beta band oscillations) has also been observed in source-resolved scalp EEG (Wagner et al. 2018).

An important question is whether a similar stopping process also operates in purely cognitive domains. Here we test whether such a mechanism is engaged to prevent unwanted memories from coming to mind. We employed the commonly-used Think/No-Think paradigm (Anderson and Green 2001), which has 3 phases. First, participants learn cue-target pairs (often word pairs). Second, they enter the Think/No-Think phase, in which they are asked to control the retrieval process. Participants perform trials in which they receive the reminder word from one of the studied pairs, presented either in Green (cuing them to retrieve and think of the associated word) or in Red (cuing them to stop retrieval). In some versions of this task participants report, after each trial, whether they experienced an intrusion of the associated memory into awareness (Levy and Anderson 2012; Benoit et al. 2015; Gagnepain et al. 2017; van Schie and Anderson 2017). These intrusion reports reveal that, despite efforts to stop retrieval, involuntary intrusions arise during No-Think trials; nevertheless, intrusions decrease with subsequent No-Think repetitions for a given pair. In a final phase, participants are asked to recall the associate for each cue word. The standard finding is that recall is worse for items that required retrieval stopping (No-Think), compared to Baseline items that were not presented in the Think/No-Think phase (Anderson and Green 2001), a phenomenon known as suppression-induced forgetting (SIF).

There are several theories of the mechanism that underlies the reduction in intrusions and the subsequent SIF effect. One prominent hypothesis is that stopping retrieval during No-Think trials engages the right-lateral prefrontal cortex to suppress hippocampal activity, disrupting retrieval and weakening the memory that is kept out of awareness (Anderson and Hanslmayr 2014; Anderson et al. 2016; Depue et al. 2006, 2016) but see other accounts (Hertel and Calcaterra 2005; Tomlinson et al. 2009; Benoit and Anderson 2012). Here we used scalp EEG and Independent Component Analysis (ICA) to test whether the same marker of right lateral prefrontal cortex stopping mechanism in the stop signal paradigm (Wagner et al. 2018), is also recruited when people have to stop memory retrieval.

Our EEG/ICA approach to testing whether the same psychological component (here “stopping”) is engaged by 2 separate tasks has been used in over a dozen papers and is described in a theoretical paper (Wessel 2018). In brief, the logic is this. If EEG data from the 2 tasks are concatenated, then passed through the ICA algorithm, ICA will decompose the data into different components that putatively relate to different psychological processes. If Task 1 involves entirely different psychological processes from Task 2, then these will be assigned to different components, and activity at a particular component for Task 1 will not show activity for Task 2. On the other hand, if Tasks 1 and 2 do engage a common psychological process then the component putatively related to that process will reveal activity for both Tasks 1 and 2. In this case, our component of interest was one with a right frontal topography, which, as described above, has been shown to relate to (motor) stopping.

Our current study very closely adheres to our pre-registered design. We planned to run 42 participants through the TNT and stop-signal paradigms with EEG. For behavior, we predicted that: (1) Final recall would be lower for No-Think items compared to baseline word pairs, replicating the standard SIF effect and (2) No-Think trials would be associated with fewer intrusions than Think trials, and the intrusions would decrease as participants practiced the No-Think task. For EEG we predicted: (1) An EEG component with a right frontal topography would show increased beta-band power for successful vs. failed stop trials (Wagner et al. 2018), (2) This same right frontal component would show increased beta-band power for No-Think trials relative to Think trials, within a short time frame after cue presentation; we specified a 300 ms latency on the assumption that such a process must be engaged quickly to countermand retrieval, and (3) During No-Think trials, the same right frontal component would show increased beta band power when retrieval is successfully stopped (Non-intrusion trials) relative to when retrieval stopping fails (intrusion trials). Validating these predictions would strongly support a common prefrontally-based mechanism for the stopping of actions and thoughts.

## Methods

### Participants

Our pre-registered study plan aimed for a sample size of 42 participants. This decision was based on: (1) a power analysis showing that 40 participants would yield 90% power to detect a standard Suppression Induced Forgetting effect with alpha = 0.05, based on η2p = 0.181, (Hellerstedt et al. 2016) and (2) the constraints required to counterbalance the assignment of word lists (see below).

Because our main analysis was focused on a right frontal “stopping” component, and because only 80–90% of participants typically show this component (Wagner et al. 2018), we anticipated a 2-step recruiting procedure: first to recruit 42 participants, then to analyze the EEG data, and then to replace those without the right frontal component with others who had one. Overall, we recruited a group of 54 right-handed participants, with a mean age of 20 (range = 18–51), composed of 39 females and 15 males. Our final analyzable sample of 41 reflected that 2 participants were excluded because of a technical failure with the EEG system, 2 for not learning at least 50% of the word pairs during the first phase of the TNT task and 2 for whom EEG recording was noisy. We replaced 7 participants who did not show the right frontal component with new ones who did.

Participants had normal/corrected to normal vision, no previous history of neurological illness or learning disabilities, no red/green color blindness, no current use of psychoactive medications and English as a primary language since early childhood (0–3 years). Our study was approved by the local IRB, #171285.

### Overview of Procedure

**Figure 1.**
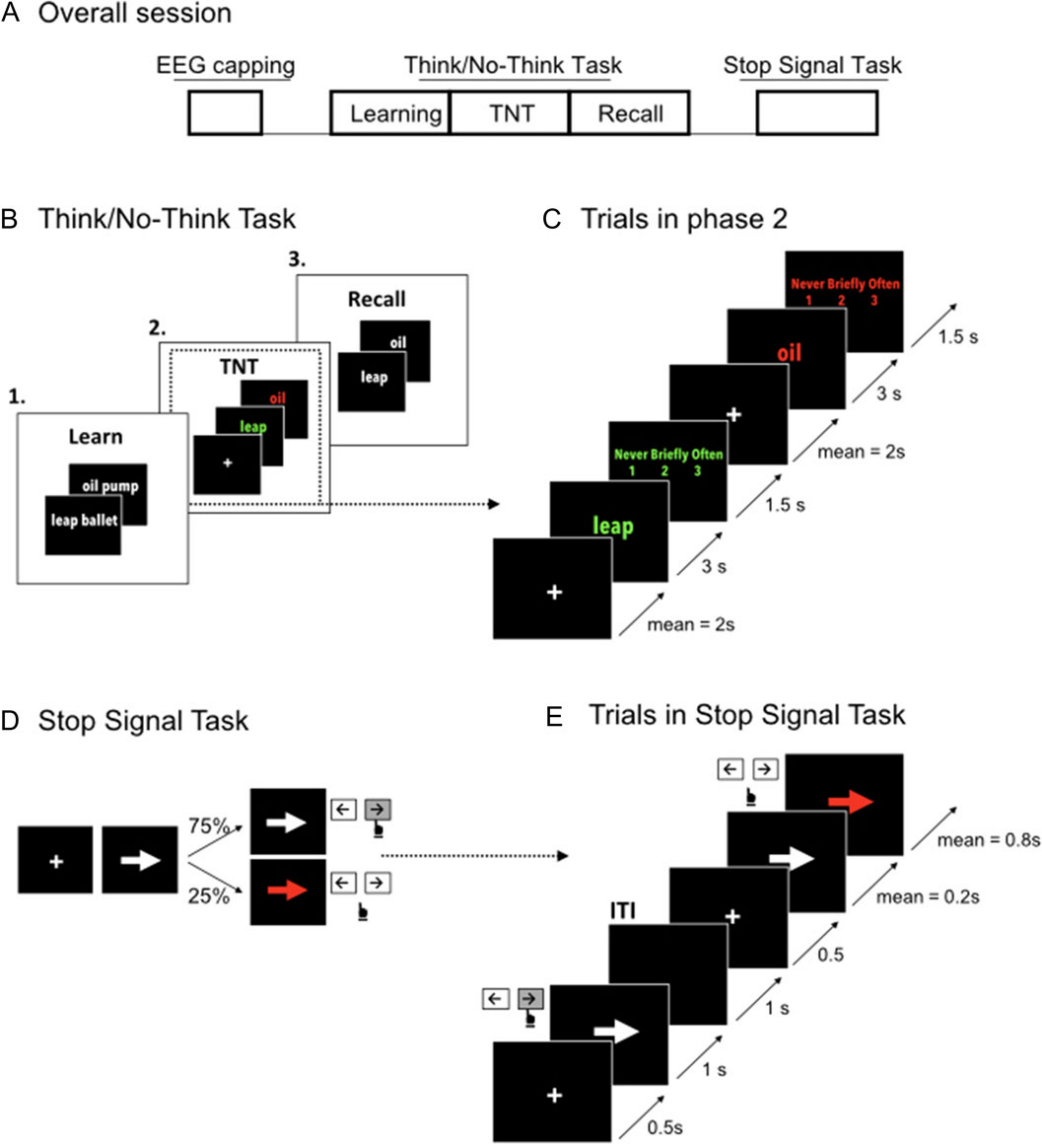
EEG recording session and the 2 tasks. (*A*) Procedure for recording EEG and behavior. (*B*) The Think/No-Think task had 3 phases. (*C*) Two illustrative trials from phase 2: Green font signaled that the subject needed to “Think” of the associated target word; red font signaled that the subject had to stop retrieval of the associate (“No-Think”). These tasks were performed in silence. After 3 s, there was an intrusion rating on each trial (subject presses a button). (*D* and *E*) Stop Signal Task. Participants initiate a button press on each trial; on 25% there is a subsequent red color change requiring them to try to stop.

After EEG-capping, participants first underwent the 3 phases of the Think/No-Think task (phase 1: learning; phase 2: Think/No-Think; phase 3: recall) followed by the Stop Signal Task (Fig. 1*A*). The tasks were run in this fixed order, TNT then SST, because the TNT was very long (over 1.5 hours, more than 2 including EEG capping) and we felt it important to have them fresh for this demanding task. Also we did not want to bias them to using a stopping strategy for the TNT task by presenting the SST first. The tasks were run using Psychtoolbox Version 3 (Brainard 1997) running on MATLAB version R2016b (Cupertino, CA). A post-experimental questionnaire was administered to assess amount of sleep participants had the night before, level of attention, compliance with instructions, and strategies used during the TNT phase.

### Think/No-Think Task

This was conducted in 3 phases (Fig. 1*B*).

#### Learning (Phase 1)

Participants learned 66 weakly related word-pairs that were taken from the University of South Florida word association norms (Nelson et al. 2004) and which were derived from stimulus sets used in prior studies (Anderson and Green 2001; Anderson et al. 2004). Of these, 18 were filler pairs for practicing the task. The 48 critical items were divided into 3 sets of 16 items that were assigned to each of the 3 conditions (Baseline, Think, and No-think), counterbalanced across participants.

Phase 1 (learning) was conducted in 2 halves, with 33 words learned per half. For the first half, participants viewed 33 word-pairs in a block-randomized order, each appearing in white font on a black background for 2 seconds. A training phase followed, in which participants were presented with the left-hand word (cue) from each pair in random order and were instructed to recall and say the matching word (target) out loud. Participants were given visual feedback on the correctness of their answer either immediately after their response or after 4 s, if no response was given. The second half proceeded identically, using the remaining 33 pairs. After this, participants were tested on recall of all 66 word pairs in block-randomized order. No feedback was given for this testing phase. Following prior work (e.g., Anderson et al. 2004), performance on this criterion test was used to classify items as officially learned or not, for the purposes of later recall analysis, see below.

Verbal responses were recorded manually by the experimenter (to give immediate feedback and evaluate test accuracy) and also via a microphone (and saved in MATLAB files for later review). Participants were required to reach a minimum of 50% correct answers to continue in the study, following past work (Anderson and Green 2001).

#### Think/No-Think (Phase 2)

Of the 48 critical items that were learned in phase 1, 16 appeared in each of the Think and No-Think conditions during Phase 2. During this phase, items were repeated 12 times each, for a total of 384 trials. These 384 trials were divided into 6 blocks of 64 trials each. In each of the 6 blocks, 2 repetitions of every Think and No-Think item were randomly presented. Before this task began, participants did 2 short practice cycles on the Think/No-Think task, using the aforementioned filler word pairs from Phase 1. After the first practice, participants completed a diagnostic questionnaire to verify that each element of the task instructions was clearly understood and applied correctly; if not, feedback was given on how to correct task performance for the next practice cycle.

Following this practice, the actual Think/No-Think task started. Each trial began with a fixation cross that appeared for an average of 1.8 s (Fig. 1*C*). Following this, the cue word (left -hand word of a given pair) appeared either in Red (No-Think condition), or in Green, (Think condition). On Think trials, participants were instructed to silently remember the matching target word (right-hand word); on No-Think trials, they were asked to suppress retrieval of the target word, preventing it from entering awareness in response to the cue. On No-Think trials, participants were instructed to use a *direct suppression* strategy to block retrieval of the target word: that is to simply focus on the cue and block the word from awareness without distracting themselves with other substitute thoughts, words or ideas. The cue remained on the screen for 3 s. After each trial, a scale asked the participant to classify their experience. Specifically, they rated, from 1 to 3 (1 = never, 2 = briefly, 3 = often) how much the target word had come into awareness on that trial.

#### Recall Phase (Phase 3)

The final recall test evaluated participants’ episodic memory for all 48 critical pairs—that is, the 16 Think and 16 No-Think word pairs as well as the 16 Baseline word pairs that had also been learned in Phase 1. Following prior work, there were 2 tests: the “Same Probe” test (SP) and the “Independent Probe” test (IP), the order of which was counterbalanced across participants. For the SP test, the cue word from each of the 66 pairs appeared in the center of the screen in white font for 4 s and the participant was asked to recall the associated target by saying it out loud. For the IP test, instead of cuing participants with the learned cue (left hand word in a pair), novel extra-list cues representing superordinate categories of the target words were shown together with the first letter of the matching target word (for example, instead of using the word “ordeal” to cue the target “roach” the IP method used the cue “insect_r”). On this latter test, participants were instructed to recall the studied right-hand word that was a member of the presented category and that began with the letter provided, and to say that target word out loud. The purpose of the IP test was to ascertain whether SIF for target items generalized to novel test cues, as would be expected if inhibitory processes suppressed the target item (Anderson & Green 2001).

### Stop Signal Task

Participants performed 5 blocks (of 100 trials each) of a standard Stop Signal Task (Fig. 1*D,E*). Each trial started with a fixation cross; after 500 ms either a leftwards or rightward pointing arrow appeared in the center of the screen and the subject tried to respond as fast as possible by pressing a left or a right button with their right index or middle finger. On approximately 25% of the trials, after the arrow appeared, a stop signal occurred (i.e., the arrow turned red)—and the participant tried to stop the response. The stop signal delay varied dynamically in 50 ms steps. Participants had 1 second to respond. The inter-trial interval was variable, depending on the response time, for a standardized trial duration of 3000 ms.

### EEG Recording and Preprocessing

We used a 64 electrode ActiveTwo system (BioSemi Instrumentation, The Netherlands) with a sampling rate of 512 Hz. The EEG electrode montage was in accordance with the 5% 10/20 system (Oostenveld and Praamstra 2001). Additional electrodes were placed on the bilateral mastoids and canthi, as well as below and above each eye. The data were on-line referenced to the BioSemi CMS-DRL reference. All offsets from the reference were kept below 25 μV.

The EEG data were high-pass filtered at 1 Hz (zero phase FIR filter, order 7500) to minimize slow drifts, and low pass filtered at 200 Hz (zero phase FIR filter, order 36). Visual inspection was used to remove EEG channels with prominent artifacts. The EEG data were then re-referenced to a common average. Visual inspection was then done to reject periods of gross artifact in the continuous EEG. The data were then partitioned into epochs of 0.5 s and those epochs containing values exceeding the average of the probability distribution of values across the data segments by 5 SD were rejected. Following this, the preprocessed data from the stop signal task and the TNT task were concatenated and submitted to ICA decomposition (Makeig et al. 1996), both to remove blink, muscle and other stereotyped artifacts and to select brain-related components of interest (see below).

### Behavioral Analysis

#### Think/No-Think (A Priori, Pre-registered)

For the final recall test (Phase 3), the main dependent variable of interest was accuracy. Following prior work, our plan was to analyze both Unconditionalized data (all word pairs included) separately from Conditionalized data (only including the word pairs initially learned, for which the test at the end of Phase 1 had shown a correct response). However, as we explain below, the Conditionalized analysis turned out to be too conservative, so we focus on the Unconditionalized henceforth. Accordingly, using accuracy from Phase 3, for each final recall test (Same Probe, SP, and Independent Probe, IP), we ran 2 separate repeated measures ANOVAs, as in prior work. The first one had the factors word pair condition (Baseline versus Think) x recall test counterbalancing (IP, SP) x counterbalancing list (A, B or C); the second one did the same except using word pair condition: Baseline versus No-Think. We added counterbalancing list as a factor to account for variance due to differences in the lists, and to provide a better test of the SIF effect.

For the analysis of intrusions (Phase 2), we followed prior work (Benoit and Anderson 2012; Hellerstedt et al. 2016) in classifying responses of “2” and “3” as intrusions and responses of “1” as non-intrusions. This was done because “3” ratings are too uncommon to be reliably analyzed. In phase 2, there were 2 repetitions for each No-Think or Think word pair per block, and 6 blocks in total, yielding 12 repetitions per pair in total. The dependent measure, for each participant, was the proportion of intrusions at each repetition (e.g., where a max of 1.0 meant that every No-Think item was accompanied by an intrusion on that repetition, and minimum of 0 meant that no No-Think item was accompanied by an intrusion at that repetition). Only pairs that were originally learned were considered in computing these scores (i.e., we analyzed intrusions, conditional on correct initial learning). An ANOVA was run with the factor repetition (i.e., 12 levels) and trial type (Think, No-Think).

In addition to examining the pattern of intrusions over repetitions, we analyzed the relationship between intrusion control and SIF. To do this, we computed an intrusion slope for each participant, to quantify how effectively participants down-regulated intrusions over repetition blocks. The intrusion scores from the 12 repetitions were grouped into 4 averages, reflecting the scores from each of 3 consecutive repetitions (e.g., average of intrusion scores for repetition 1–3, 4–6, 7–9 and 10–12). We then computed the slope of these 4 averages. To account for the fact that the magnitude of the slope is constrained by the level of intrusions on the first trial, we proportionalized the slope by dividing it by the first of the 4 averages (the average from repetition1-3) (Levy and Anderson 2012). Intrusion slopes were compared through bootstrapped robust correlation (Pernet et al. 2012) against a) z-normalized SIF (the number of items recalled for No-Think versus Baseline pairs) separately for Same Probe and Independent Probe tests, and merged SP and IP, and b) SSRT from the stop signal task (see below).

#### Think/No-Think (Post hoc)

In line with the previous literature (Schmitz et al. 2017), we also conducted a post hoc Pearson’s Skipped correlation (Pernet et al. 2012) between z-normalized SIF (Baseline recall − No-Think recall) and SSRT. This latter analysis was performed separately using the SIF for the Same Probe test, the Independent Probe test, and the merged SP and IP tests (overall SIF effect). Prior work suggests that the strongest relationship between SSRT and SIF should arise on the Independent Probe test, which is less influenced by non-inhibitory sources of forgetting (Anderson and Levy 2007; Levy et al. 2007; Anderson and Hanslmayr 2014; Schilling et al. 2014).

Finally, we calculated how many of the word pairs that were excluded in the Conditionalized data (because they were classified as “not learned” in the learning phase 1) were in fact not remembered in the final recall tests; it is possible that some word-pairs were not correctly reported during the criterion test of learning phase 1 due to distraction/lack of time (and therefore marked as “not-learned”), and that they were, in fact, learned.

#### Stop Signal Task

We calculated mean correct Go RT, mean failed stop RT, p(stop), number of discrimination and omission errors on go trials, and mean SSD. The SSRT was calculated using the mean method (Verbruggen and Logan 2009). RTs for correct Go trials was compared against Failed Stop to test the independence assumption of the race model.

### EEG Analysis

The specific planned analyses described below were pre-registered. We used EEGLAB 14.1.1b (Delorme and Makeig 2004) running in MATLAB2015b (The MathWorks).

#### Preparing the Independent Components Analysis

**Figure 2.**
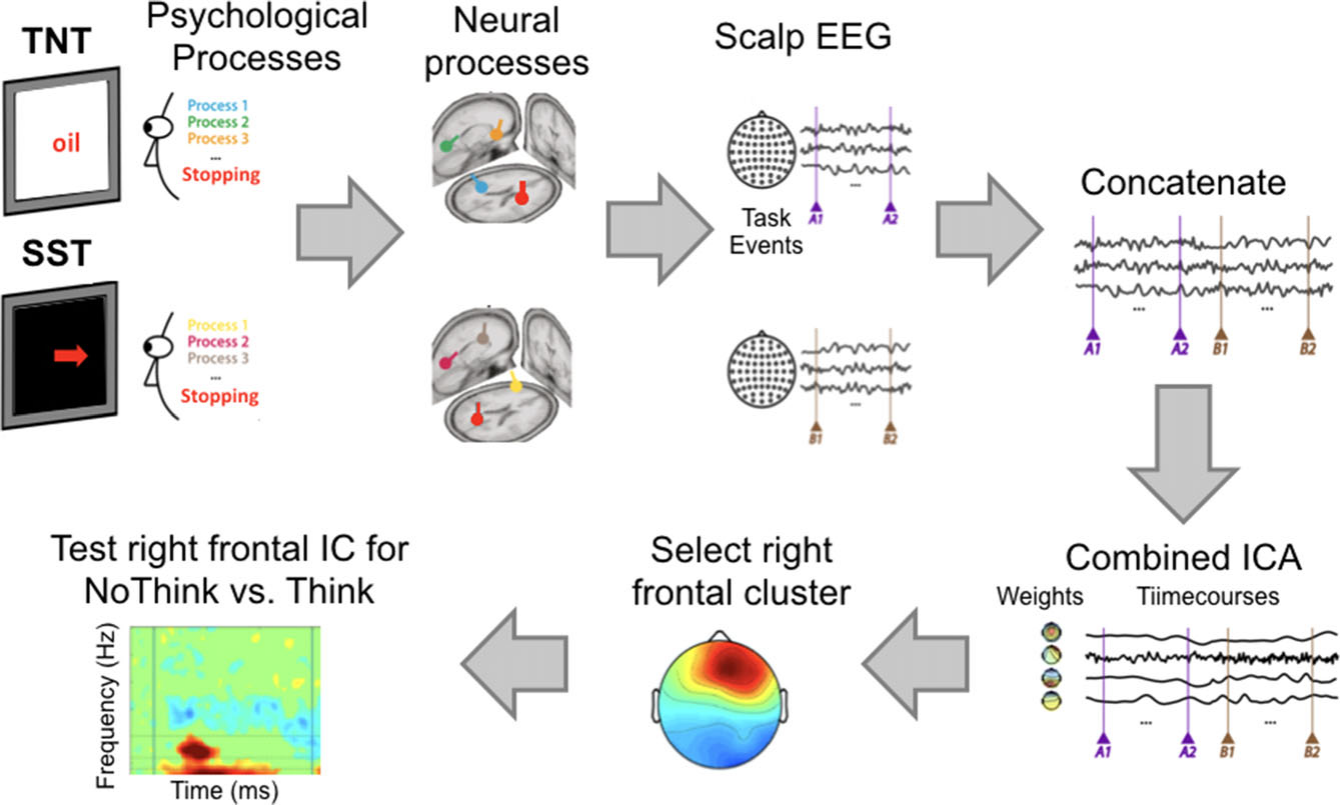
Using Independent Components Analysis to test if 2 tasks engage the same process. Each task involves psychological processes which map to neural circuits/sources and generate EEG activity at the scalp. The EEG data from the 2 tasks (done in the order TNT then Stop Signal) are concatenated and then submitted to ICA in each participant separately. Automatic clustering is then done on the components to identify clusters across participants. Most participants contribute to a right frontal cluster—which we have earlier shown to index action-stopping (Wagner et al. 2018). The main hypothesis test is then to assess whether and when there is a power-increase in this right frontal component for No-Think vs. Think trials. Figure adapted from Wessel (2018).

We adhere to the exact approach intended in the pre-registered document, and which has been described in detail in our earlier publication, and for which we here provide scripts (Wagner et al. 2018) (Fig. 2). Here we describe it anew:

- We concatenated the preprocessed EEG data from the Think/No-Think task and the stop signal task for each participant.
- The concatenated EEG data were submitted to extended Infomax ICA (Bell and Sejnowski 1995; Makeig et al. 1996) for each participant, resulting in as many components as channels.
- Non-brain components were then rejected. We did this by visually inspecting each Independent Component based on, event-locked time courses, mean power spectra, dipoles located within brain and explaining at least 85% of the variance in the scalp map. Equivalent current dipoles were estimated using a standardized 3-shell boundary element head model implemented in the DIPFIT toolbox within EEGLAB (sccn.ucsd.edu/eeglab) (Oostenveld and Oostendorp 2002; Delorme et al. 2012). Standard electrode locations corresponding to the Extended 10–20 System were aligned with a standard brain model (MNI).
- Clustering. The challenge is to identify similar components across participants (here we had aimed for 42 participants to have a similar component). Clustering is done on feature vectors from each participants’ data. These features were: 1) IC dipole locations, 2) scalp projection, 3) power spectra in the range 3–200 Hz, 4) event related potentials in the time window 0–600 ms following the stop signal), and 5) event-related spectral perturbations (ERSPs) in the frequency range from 3 to 20 Hz and time window from 0 to 600 ms following the stop signal. Note that the clustering was only run on successful stop trials obtained from the stop signal task. The rationale was to identify a cluster with a right frontal topography showing a fast beta power increase relative to the stop signal before SSRT. Once the clustering was complete we moved to hypothesis-testing, described below.

We now describe, in more detail, the process of preparing the EEG data for clustering. First, pre-clustering dimensionality reduction. We selected the relevant parts of each feature. For example for the ERP we selected the mean over trials 0–600 ms after the stop signal which resulted in ~307 data points (given the sampling rate of 512 Hz). We then reduced these 307 points to 10 dimensions using principal component analysis (PCA). PCA finds orthogonal subspaces that explain maximal variance of the data, with the first principal component explaining the largest part of the variance of the data. We applied the same procedure to the spectra, ERSP and scalp maps. Dipoles have inherently only 3 dimensions (the tailarach coordinates x,y,z). We thus ended up with 5 feature vectors, 4 each with 10 dimensions and 1 with 3 dimensions (related to dipoles) for each IC.

Second, the weighting of feature vectors. The dimensionally reduced feature vectors were then weighted for subsequent clustering i.e., dipole locations: weight 12; scalp projection: weight 4; power spectra: weight 3; event-related potentials: weight 3; and event related spectral perturbations (ERSPs): weight 10. These weights were chosen based on our hypothesis: we expected a beta band increase (13–20 Hz) with a right frontal scalp distribution and a timing from 0 to 600 ms after the stop signal. We also know that an ERP occurs at around 300 ms following the stop signal. The relatively large weight for the ERSPs made clustering similarly sensitive to IC ERSP differences as for dipole location.

Third, concatenation and clustering. The 5 feature vectors were then concatenated for each IC and further reduced to 10 principal components using PCA. We then ran k-means clustering (*k* = 15).

Our main planned analysis focused on a cluster with a right frontal topography based on our extant work on action-stopping (Wagner et al. 2018). As explained in participant recruitment above, of our initial sample of 42 participants, 35 initially contributed an IC to this cluster, therefore we added another 7, of which 6 contributed an IC to the right frontal cluster for a final sample of *N* = 41.

A secondary planned analysis focused on a cluster with a dorsomedial topography, for which the P3 onset latency has been shown to be earlier for successful vs. failed stop trials (Wessel and Aron 2015). Clustering here was again done exclusively on successful stop trials.

#### A Priori Hypothesis Testing

For the IC cluster with the right lateralized topography, based on (Wagner et al. 2018), we ran several analyses using Event-Related Spectral Perturbations (ERSPs):

- The stop signal task. The data were segmented into time epochs relative to onsets of the stop signal (i.e., from −2 to 1.5 s around the stop signal). We generated ERSPs for successful stop trials, failed stop trials and the difference. Relative changes in spectral power were obtained by computing the mean difference between each single-trial log spectrogram and the mean baseline spectrum (the average log spectrum between −2 and −1 preceding the stop signal—this baseline was chosen since it falls into the inter trial interval when no cues occur - the go cue occurred around 100 to 500 ms before the stop signal). Significant deviations from the baseline were detected using a nonparametric bootstrap approach (Delorme and Makeig 2004) and corrected for false discovery rate (Benjamini et al. 2001) with a significance level set a priori at 0.05. We generated ERSPs for Successful and Failed Stop trials, and the difference. Note that the clustering method simply uses beta power as one out of 5 features to try to assign individual participants’ components to an overall cluster; it does not select a subset of trials with relatively increased beta, or beta at a time-frame before SSRT. Thus selecting components (across participants) based on successful stop trials alone does not bias a result to find increased right frontal beta before SSRT on successful stop trials, or a successful vs. failed difference.
- The Think vs. No-Think comparison. The data were segmented into time epochs relative to onsets of the Think/No-Think cue (i.e., from −1 s to 1.5 s around the Think/No-think cue). We generated ERSPs for the Think and No-Think trials and the difference, time locked to the cue. We used a baseline between −1 to 0 sec preceding the Think/No-think cue, and the same approach for detecting significant differences from baseline as for the stop signal data.
- The No-Think intrusion analysis. The data were segmented into time epochs relative to onsets of the No-Think cue (i.e., from −1 s to 1.5 s around cue). We generated ERSPs for No-Think non-intrusion, No-Think intrusion, and the difference. We used a baseline between −1 to 0 sec preceding the No-think cue. Because there was an unequal number of trials rated as intrusions/non-intrusions for the No-Think trials, we used a bootstrap approach within each subject to draw subsequent random samples of the same number of trials from intrusion and non-intrusion trials to construct ERSP images.

For the IC cluster with the dorsomedial topography 2 analyses were run:

- The stop signal task. We aimed to verify, for the ERP from this component, that the P3 onset latency was earlier for successful vs. failed stop trials, as in (Wessel and Aron 2015).
- The Think/No-Think task. We generated ERSPs for No-Think and Think trials, and the difference.

#### Other Planned Analyses

We planned to test whether the change in intrusion ratings across time in phase 2 related to the stopping process. Accordingly, for each “pixel” of time-frequency space we ran a linear regression of proportionalized intrusion reduction against the ERSP activity in the right frontal stopping component for No-Think trials in phase 2.

### Multiple Comparisons

For the TNT task, the planned behavioral analyses consist of 4 ANOVAs for accuracy, an ANOVA for intrusions and 4 correlations for the intrusion slope analysis. Significance testing used alpha = 0.05 without any correction. All these outcomes were hypothesis-driven based on extant results, and we wanted to strike the correct balance between Type 1 and Type 2 error. The stop signal task behavioral results are not statistical tests, but merely metrics of task performance. The EEG results concern ERSPs for conditions vs. baseline, or between-condition comparisons. All were planned, and each ERSP plot was individually corrected for multiple comparisions across all time-frequency points with FDR < 0.05.

## Results

### Behavior

#### Think/No-Think task

Our study plan for the recall phase specified doing both Conditionalized analyses (i.e., only on the word pairs that were learned by the end of Phase 1) and Unconditionalized analyses (all word pairs). However, a closer look at the Conditionalized analysis showed that out of an average of 5 “not-learned” word-pairs in Phase 1 (s.d. = 3.43) that were excluded from the Conditionalized analyses, 2.76 word pairs (more than 50%) were actually remembered correctly in Phase 3 in either the Same Probe test, the Independent Probe test or both tests (s.d. = 1.91). This suggests that the conditionalization procedure excluded many items that were truly learned by participants, together with truly unlearned items. Because the rate of learned-item exclusion is particularly high in this sample, we only focus further analyses on unconditionalized data to include as many of the usable trials as possible, which was especially relevant to have more power for the EEG analysis. Here, we start by reporting behavioral results for Phase 3 (recall) for SP and IP tests, and then for Intrusions in Phase 2.

**Figure 3.**
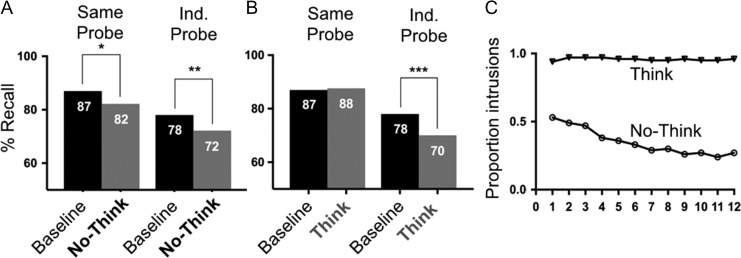
Behavioral results. (*A*) Recall results from Phase 3. Left panel, Baseline vs. No-Think; right panel, Baseline vs. Think. (*B*) Results from the trial-by-trial intrusion rating in phase 2. Each cue was repeated 12 times.

For the same probe, ANOVA for Baseline and No-Think items revealed a main effect of condition, *F*(1,35) = 4.449, *P* = 0.042, partial eta sq. = 0.113 (Fig. 3*A*). Planned comparisons showed a significant reduction in final recall for No-Think (M = 82.5%, SEM = 0.026) vs. Baseline (M = 87%, SEM = 0.018), *t*(41) = 2.136, *P* = 0.042. For the ANOVA comparing Baseline and Think items, there was no main effect of condition.

For the independent probe, the ANOVA for Baseline and No-Think items showed a main effect of condition, *F*(1,35) = 8.469, *P* = 0.006, partial eta sq. = 0.195, and a significant interaction between condition and counterbalancing list (Fig. 3*B*). Planned comparisons showed a significant reduction in final recall for No-Think (M = 72%, SEM = 0.19) vs. Baseline (M = 78%, SEM = 0.19), *t(*41) = 2.954, *P* = 0.006. For the ANOVA comparing Baseline and Think items, there was a main effect of condition, *F*(1,35) = 16.246, *P* < 0.001, partial eta sq. = 0.317. Planned comparisons showed a significant decrease in final recall of Think (M = 70%, SEM = 0.17) vs. Baseline, *t*(41) = 4.1, *P* < 0.001.

For the intrusion analysis, we calculated the proportion of trials on which participants reported awareness of the associated target separately for No-Think and Think items, at each repetition. We ran a 2-way ANOVA with the factors repetition (0–12 repetitions) and Task (No-Think vs Think) and found an extremely robust main effect of Task, *F*(1,40) = 291.93, *P* < 0.001, partial eta sq. = 0.879, and a significant interaction between repetition and Task *F*(7269) = 23.38, *P* < 0.001. Whereas intrusions significantly decreased across the 12 repetitions during the No-Think trials, they remained at a constant and high level across the 12 repetitions of the Think trials (Fig. 3*C*).

In summary, these suppression-induced forgetting (SIF) and intrusion findings indicate highly successful control over the retrieval process during No-Think trials, and largely replicate prior findings (Levy and Anderson 2012; Benoit et al. 2015; van Schie and Anderson 2017).

We then tested if individual differences in the No-Think intrusion slope related to subsequent forgetting (i.e., the SIF effect) and also to the speed of stopping (SSRT). The correlation between No-Think intrusion slope and SIF was not significant for either the Same Probe, SP: *r *= −0.06, nor the Independent Probe: IP *r* = 0.05, nor the merged SP and IP: *r* = 0.02. Similarly, no correlation was found between No-Think intrusion slope and SSRT: *r* = 0.05.

#### Post hoc

We tested if those participants with more SIF (z-normalized values) had faster SSRT. This was done for SP, IP, and merged SP and IP (i.e., 3 correlations). There was a negative correlation of SSRT with SIF for the merged same probe and independent probe tests—suggesting, as expected, that those participants with a higher SIF effect had faster SSRT (*r* = −0.115), but this was not significant C.I. [−0.417 0.255]. However, this relationship was marginally reliable when considering SIF on the IP test in isolation (*r* = −0.257, *P* = 0.096 in the unconditionalized data), consistent with the proposed greater sensitivity of this test to inhibitory control (Anderson and Levy 2007; Schilling et al. 2014).

#### Stop Signal Task

The metrics were typical for healthy young participants. Go RT was slower than failed stop RT in every participant, consistent with the race model (go RT = 534 ms, failed stop RT = 460). The percentage of successful stopping was 51% on average and there were few errors on go trials (~2%). The stop signal delay that gave ~50% stop rate was 265 ms and mean SSRT was 268 ms.

### EEG

#### A Priori Analysis

**Figure 4.**
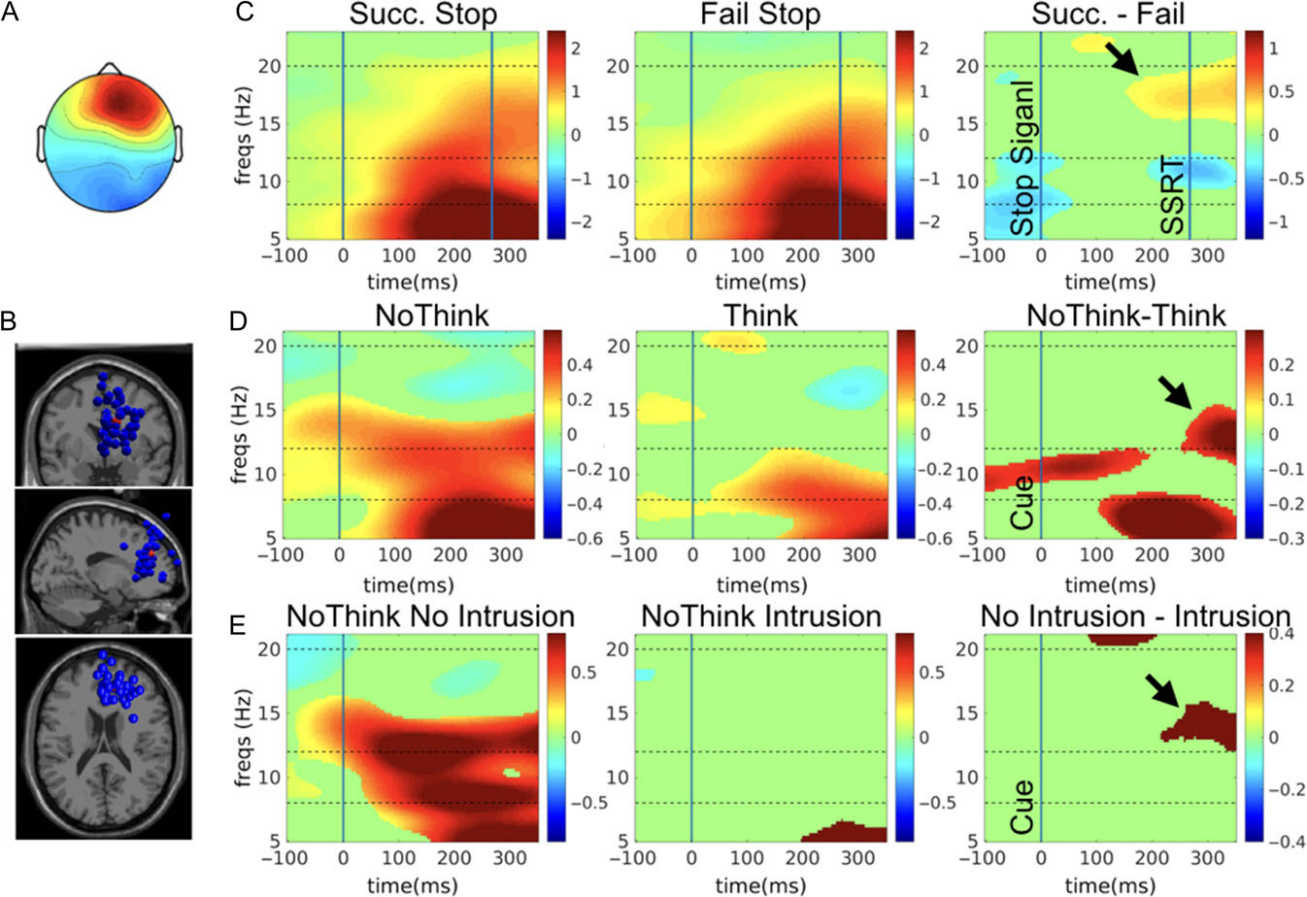
Event-related spectral perturbations for the right frontal component. (*A*) The right frontal topography representing the mean scalp projection map of automatically clustered right frontal components from 41 participants. (*B*) The estimated equivalent dipole locations of the independent component in each participant (blue spheres) and their centroid (red sphere) visualized in the MNI template brain. (*C*) ERSP analysis for the stop signal task. There is increased right frontal beta band power for successful vs. failed stop trials before SSRT. (*D*) ERSP for No-Think vs. Think. There is increased early right frontal beta power for No-Think vs. Think trials. (*E*) ERSP for intrusion analysis on No-Think trials. There is increased early right frontal beta power for No-Think trials without an intrusion. All maps are masked for significance (*P* < 0.05) and FDR-corrected for multiple comparisons. The units of power are dB.

After ICA was run on the merged Think/No-Think and stop signal data for each participant, and after automatic clustering was done, we identified a right frontal cluster to which 41 participants contributed an independent component (Fig. 4*A,B*).

ERSP analysis of Successful Stop Trials, Failed Stop trials and the difference was consistent with our earlier studies (Wagner et al. 2018). Specifically, we observed that for successful stop trials, there was a power increase across several bands, before SSRT, and that this was significantly greater in the beta band compared to failed stop trials before SSRT (Fig. 4*C*). This validates our typical right frontal beta-band stopping signature in this study.

We next analyzed the same right frontal cluster for the Think/No-Think phase of the study. Strikingly, there was also an early increase of power for No-Think trials more than for Think trials in the beta band (in the sub 500 ms time-scale) (Fig. 4*D*). This validates a core prediction of the study – that a brain signature for action stopping is also elicited on No-Think trials.

Turning to the Intrusion analysis, for No-Think trials that were not accompanied by intrusions (Non-Intrusions), there was also an early increase of power and this was greater compared to No-Think trials with Intrusions, again in the sub 500 ms time-scale (Fig. 4*E*).

To test whether the change in intrusion rating over repetitions in phase 2 related to the stopping process, we ran a linear regression of proportionalized intrusion reduction against the ERSP activity in the right frontal stopping component for No-Think trials. There was no significant activity for this contrast.

**Figure 5.**
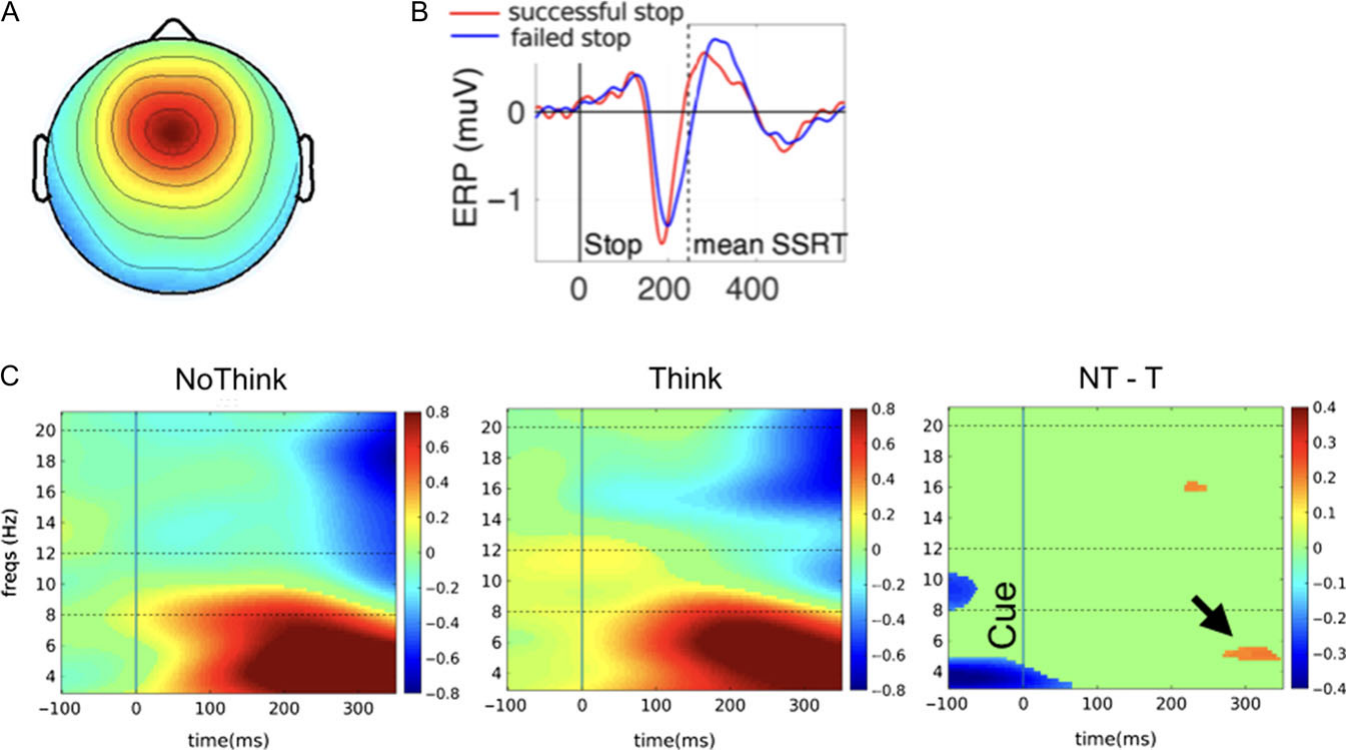
EEG results for the dorsomedial component. (*A*) The dorsomedial topography representing the cluster mean scalp projection map of automatically clustered dorsomedial components from 36 participants. (*B*) ERP analysis from this cluster shows the P3 onset latency is earlier for successful vs. failed stop trials, c.f. (Wessel and Aron 2015). (*C*) ERSP analysis shows there is increased low frequency power for No-Think vs. Think for this component. The plots are FDR corrected for multiple comparisons. Power units are in dB.

For the dorsomedial cluster, the mean scalp topography averaged over the components for all 36 participants is shown in Figure 5*A*. For the stop signal analysis, and consistent with published reports, there was an earlier P3 onset latency for successful vs. failed stop trials (Fig. 5*B*) (Wessel and Aron 2015; Dutra et al. 2018). Turning to the NoThink and Think trials, ERSP analysis of this component showed there was significant low frequency activity for No-Think trials and this was significantly greater compared to Think in the theta band (4–8 Hz) within about 300 ms (Fig. 5*C*). This confirms that No-Think trials also engaged this other component of the stopping process.

## Discussion

Prior work has established a distinctive right frontal beta signature that relates to successful action stopping. Here, we tested whether this same signature is also observed when people try to stop a strictly cognitive, non-motor process: i.e., memory retrieval. We recorded EEG while participants performed the Think/No-Think and Stop Signal Tasks. Behavioral findings largely replicated prior work. For the Think/No-Think task, we found: a) the typical pattern of suppression-induced forgetting (SIF) arising from participants’ efforts to stop the retrieval process (e.g., Anderson and Green 2001; Bergstrom et al. 2009; Depue et al. 2013), and b) a decline in intrusions of No-Think items over repeated control attempts (Levy and Anderson 2012). For the Stop-Signal task, behavioral performance was typical and EEG analysis validated the signature of increased right frontal beta-band power for successful vs. failed stop trials, before SSRT (Wagner et al. 2018). Importantly, for the Think/No-Think task, we observed a similar increase in right frontal beta-band power during No-Think trials (when participants sought to stop the retrieval process), more so than on Think trials, when they did not. Strikingly, this early right frontal beta effect (beginning ~300 ms after the No-Think cue, as we had predicted in our pre-registered document) was more pronounced during No-Think trials in which retrieval was successfully stopped (i.e., Non-Intrusion trials) compared to No-Think trials *with* intrusions. Finally, a secondary planned focus of the study was on a dorsomedial component for which prior studies revealed an ERP stopping signature—an earlier P3 onset latency for successful vs. failed stop trials – as well as low frequency power increases (Wessel and Aron 2015; Dutra et al. 2018). Here too, we observed the same ERP result on action stopping trials, and also found increased early activity in this putative stopping component for No-Think vs. Think trials in low frequency (theta) band, as shown by Depue et al. (2013).

These results provide novel information about the electrophysiology of preventing memory retrieval, c.f. (Mecklinger et al. 2009; Chen et al. 2012; Hanslmayr et al. 2009, 2012; Waldhauser et al. 2015; Hellerstedt et al. 2016). More specifically, they confirm our prediction that brain signatures for rapid action stopping are also recruited during the stopping of episodic retrieval, within just a few hundred milliseconds of cue onset. This provides important converging evidence for an executive control theory concerning the prevention of intrusive memories (Anderson and Green 2001; Anderson and Hanslmayr 2014; Anderson et al. 2016) motivated in part by fMRI studies (Banich et al. 2009; Depue et al. 2016). A meta-analysis of fMRI studies has shown greater engagement of right lateral frontal cortex during No-Think vs. Think trials, which overlaps with motor stopping activations (stop > go) observed in the stop signal task (Guo et al. 2018). Overlap in fMRI activation across tasks, however, is not definitive evidence for a common process (even if predicted in advance based on theory), because a particular brain region can contribute to many different processes (Poldrack 2006). By contrast, we argue that time-frequency analysis of EEG provides greater specificity (across all of spatial topography, frequency and time) and this permits increased confidence in a common underlying process. Specifically, No-Think vs. Think trials elicited increased activity in a right frontal spatial component, within the beta band (13 to 20 Hz), within less than 300 ms. This right frontal signature is similar to the one seen during action stopping in the Stop Signal task in scalp EEG in this study, and other such studies (Wagner et al. 2018), and also from intracranial EEG recordings from the right inferior frontal cortex (Swann et al. 2009; Wessel et al. 2013). Moreover, disruption studies have shown the right IFC is critical for action-stopping (Aron et al. 2014). We propose therefore, that these findings support the view that preventing inappropriate memory retrievals also recruits a right lateral frontal cortex stopping process. We warrant, however, that further research is needed to confirm this – for example, using causal approaches such as Transcranial Magnetic Stimulation

The logic of the 2-task ICA approach we used here is as follows (and see Wessel 2018). First, if Successful action-stopping and No-Think trials recruit *different* psychological processes then ICA will disentangle these, and if one then identifies a right frontal cluster (spatial filter) for action-stopping, there will be *no activity* within this spatial filter on the No-Think trials. By contrast, if one *does detect* activity on No-Think trials then that is evidence that there is a common process between action-stopping and Not Thinking. It has been argued that this common “process identification” procedure is considerably more powerful, logically, than a “localizer” approach in which one merely uses a spatial filter from one task to test activity in the other (and see Wessel 2018). Note that activity for No-Think (TNT task) does not necessarily have to occur in the same frequency band as action stopping (SST) for this claim to be supported (see e.g., Wessel and Aron 2014). In fact, here we show that all of Successful vs. Failed stop, No-Think vs. Think, and No-Think No-intrusion vs. No-Think Intrusion contrasts have activity differences in the right frontal component and for a similar time period and *in the beta band*. Figure 4*C* shows that the significant difference for Successful vs. Failed Stop is, in the group map, about 15 to 20 Hz, while for No-Think vs. Think it is significant in the range of about 12 to 15 Hz (also for No-Think No-Intrusion vs. No-Think Intrusion). Thus, both stopping action and stopping memory retrieval recruit beta power within 300 ms—albeit not in the exact same frequency range. The reason for this discrepancy is unclear. One possibility is that increased beta power for action-stopping and memory-stopping do reflect the same process, prefrontal inhibitory control, but not via the exact same circuitry—for example, it has been suggested that higher beta frequency vs. lower beta frequency relate to hyperdirect vs. indirect cortical-basal ganglia pathways (Jenkinson and Brown 2011).

We considered whether increased beta power for action-stopping and No-Think trials reflect a common process (across tasks) that is *not* inhibitory control, such as attention, difficulty or arousal. Several considerations speak against this. First, ECoG recording in a sizeable sample of patients showed similar broad-band *gamma* activity (50–150 Hz) for Successful vs. Failed stop trials before SSRT in the right frontal pars opercularis region (Bartoli et al. 2018). We suppose this reflects equivalent triggering of the stopping process on both types of trials. By contrast to those gamma findings, ECoG activity in the beta frequency range (13–20 Hz) *does* show greater power for successful vs. failed trials before SSRT (Swann et al. 2009; Wessel et al. 2013), as it does here in scalp EEG. Based on these observations, we suppose that the beta band reflects a network property of the frontal-basal ganglia system (Swann et al. 2011) that is stronger on successful stop trials where inhibitory control successfully intercepts the Go process [rather than reflecting arousal, attention or difficulty]. Second, and consistent with the above, in the action-stopping case, the beta band difference for successful vs. failed stopping is also evident in the basal ganglia (Zavala et al. 2015; Aron et al. 2016)—and, in the motor domain, increased beta band power is thought to reflect an akinetic state (Engel and Fries 2010). Third, what also speaks against mere differences in attention is that there was an early beta power difference for No-Think vs. Think even though they had matched proportions and the only thing differentiating them was cue color. Moreover, this early beta effect was greater for those No-Think trials without an intrusion vs. those with an intrusion (even though the stimulus is a word written in red font in both cases). We argue these considerations point to a common stopping process rather than attention, difficulty or arousal. However more research is clearly needed to better understand the right frontal, above-baseline, increases in beta power in such tasks, and to better disentangle detection/attention and response control. As noted above, further work is clearly needed to test the causal role of this right frontal region (and putative subcortical network) using methods such as Transcranial Magnetic Stimulation. Validating a causal role would strongly support the theory that the SIF effect owes to a prefrontal suppression system, and against alternative accounts such as associative interference. Behavioral relationships converge with this possibility: faster SSRTs were associated with greater suppression-induced forgetting on the independent probe test of forgetting (marginally significant); with this independent probe best having been shown to be more diagnostic of the involvement of inhibition (Anderson and Green 2001) and see for discussion Anderson and Levy (2007) and Schilling et al. (2014). However, again, more research is clearly needed to better understand the right frontal, above-baseline, increases in beta power in such tasks, and to better disentangle detection/attention and response control.

Assuming the right frontal beta signature is indeed one of stopping on No-Think trials, how could this induce forgetting and prevent intrusions? We consider several possibilities. First, the beta signature may be unrelated to stopping retrieval and forgetting per se and might simply reflect the stopping of motor aspects of the Think/No-Think task. For example, in this task, participants might have to stop the speech (motoric) aspect of the target word which has nothing to do with memory (on this account, the forgetting is generated by other processes). Yet here the prepotency of speech production was quite low, given that the entire 30 to 45-min task was performed silently, with no speech requirement; and even if we entertained that covert generation of words required stopping of speech, this demand is arguably greater in the Think condition. Also, there was a difference in frontal beta power for No-Think trials without intrusions vs. those with intrusions, even though the latter should require greater stopping of speech. Yet we warrant that the relation to forgetting is indirect here and future research is needed to better flesh out whether the right frontal signature relates to this. A second explanation for how the putative prefrontal stopping process relates to forgetting is that it reflects a prefrontal shut-down of the medial temporal lobe, reviewed by Anderson et al. (2016). On this view, whereas action-stopping putatively requires a right lateral PFC-basal-ganglia-M1 network, a memory-stopping system putatively requires a right lateral PFC-MTL network; different modes, but a common frontal signature. This account may be testable by showing that changes in the prefrontal beta signature (perhaps induced by brain stimulation) triggers changes in medial temporal lobe activity (recorded with fMRI or LFPs). A final possibility is that the prefrontally-mediated stop process controls the entrance of retrieved contents into working memory (c.f. Badre and Wagner 2007) (Scimeca and Badre 2012). For example, during No-Think trials, this process may operate via the basal ganglia in a way that impacts a thalamocortical retrieval process (see, e.g., Guo et al. 2018 for evidence for engagement of the Basal Ganglia during retrieval suppression). On this view, when the cue occurs on No-Think trials, pattern completion via the medial temporal lobe begins for the target, but this has to then trigger reinstatement in neocortex to achieve recollection. The stopping process may interfere with this latter reinstatement aspect of retrieval. We note that whereas this explains how intrusions might be prevented, it is not clear how this would explain SIF. These accounts of how stopping processes may prevent recall are not mutually exclusive, and successful control of retrieval may entail stopping at various points in the retrieval process.

Whereas the study confirmed most of our pre-registered predictions, some findings were not expected. First, on the behavioral level, whereas several studies have found a relationship between the ability to reduce intrusions over repeated suppression attempts and later suppression-induced forgetting (Levy and Anderson 2012; Hellerstedt et al. 2016; Guo et al. 2018), the current study found no significant relationship between these metrics. It is unclear why this relationship was not found. As noted in the preceding discussion, however, recall can be stopped by interrupting mechanisms at any stage of the retrieval process, only some of which, when stopped, may give rise to forgetting. One speculation is that if intrusions are sometimes controlled in ways that do not require hippocampal suppression, this relationship between intrusion control and forgetting may not emerge. This possibility may also account for why the ability to reduce intrusions over blocks was not correlated with the frontal beta component. Second, whereas many studies using the Think/No-Think show that, in the final recall test, recall for Think items is above-baseline (due ostensibly to retrieval practice in the earlier Think/No-Think phase), evidence for such retrieval-based facilitation was lacking in the present study. On the Same Probe test, there was no facilitation for Think items, and on the independent probe test, recall performance for Think items was actually below baseline. One factor likely to have contributed to the lack of facilitation on the Same Probe test is the near ceiling level performance observed on that test, and the consequent reduction in sensitivity to the benefits of retrieval during Think trials. The reduced recall for Think items on the Independent Probe test has also been observed in other studies with this paradigm (e.g., Paz-Alonso et al. 2009). This effect has been attributed to encoding specificity effects driven by the repeated retrieval of No-Think items in response to studied cues (see, e.g., Paz-Alonso et al. 2009). Finally, we note that, as in previous studies of action-stopping (Wagner et al. 2018), about 10–20% of participants (here 14%) did not show the right frontal stopping component. We suppose this owes to differences in cortical folding which is known to affect the detection of the local field potential (Luck 2014). In any event, our pre-registered study plan had anticipated such attrition, and adjusted for it, and we here analyzed 41 participants with a right frontal component, very close to our planned sample size of 42.

In conclusion, we show that the requirement to prevent a thought from coming to mind quickly recruits a similar EEG signature as stopping action. This EEG signature – a right frontal beta band increase that occurs within 300 ms – could be a specific neural marker of stopping via right lateral prefrontal recruitment (perhaps specifically the rIFC), and also from basal ganglia (Aron et al. 2016). The specificity of this signature points strongly to the possibility of a “thought stopping” mechanism and is consistent with the few extant reports in this area (Chiu and Egner 2015a,b; Depue et al. 2016; Guo et al. 2018) and in so doing delivers on the central “thought-action linking” premise of the Logan and Cowan (1984) seminal paper entitled “On the ability to inhibition thought [sic] and action”. Building this link to action-stopping opens the prospect of leveraging the substantial across-species knowledge of the neural basis of stopping (Schall and Godlove 2012; Bari and Robbins 2013; Jahanshahi et al. 2015; Schmidt and Berke 2017) into the domain of long-term memory retrieval. This could have important implications for better understanding basal ganglia “gating” of prefrontal cortex (Scimeca and Badre 2012), for prefrontal—medial temporal lobe interactions (Anderson et al. 2016), for better understanding disorders of rumination and anxiety (Banich et al. 2009; Munakata et al. 2011; Sacchet et al. 2017) and how the mind is more generally “cleared” (Banich et al. 2015; Lewis-Peacock et al. 2018).

## Notes

The pre-registered plan (uploaded on 10-27-17), the artifact rejected EEG data (32GB), scripts to generate the core EEG results, and the Behavioral files can be found at the Open Science Framework https://osf.io/2ufmz/
